# LIFEHOUSE’s Functional Nutrition Examination (Physical Exam, Anthropometrics, and Selected Biomarkers) Informs Personalized Wellness Interventions

**DOI:** 10.3390/jpm13040594

**Published:** 2023-03-28

**Authors:** Michael Stone, Dan Lukaczer, Christopher R. D’Adamo, Nicole Dotson, Andrey Volkov, Deanna Minich, Dina Metti, Michelle Leary, Monique Class, Malisa Carullo, Erik Lundquist, Brent Eck, Jose Ordovas, Joseph Lamb, Jeffrey Bland

**Affiliations:** 1 Office of Personalized Health and Well-Being, Medical College of Georgia, AU/UGA Medical Partnership, Athens, GA 30606, USA; 2 Institute for Functional Medicine, Federal Way, WA 98003, USA; 3 Personalized Lifestyle Medicine Center, Gig Harbor, WA 98332, USA; 4 Center for Integrative Medicine, University of Maryland, Baltimore, MD 21201, USA; 5 Bennett Data Sciences, San Diego, CA 92107, USA; 6 Human Nutrition and Functional Medicine, University of Western States, Portland, OR 97213, USA; 7 Swedish Medical Group, Seattle, WA 98122, USA; 8 Vida Integrated Health, Seattle, WA 98112, USA; 9 The Center for Functional Medicine, Stamford, CT 06905, USA; 10 Metagenics, Inc., Aliso Viejo, CA 92656, USA; 11 Personalized Lifestyle Medicine Center, Aliso Viejo, CA 92656, USA; 12 Jean Meyer USDA Human Nutrition Center on Aging, Tufts University, Boston, MA 02111, USA; 13 Personalized Lifestyle Medicine Institute, Bainbridge Island, WA 98110, USA

**Keywords:** nutrients, nutrient insufficiency, nutrient deficiency, physical examination, functional medicine, personalized lifestyle medicine, biomarkers, ‘N-of-1’ trials, pattern recognition, LIFEHOUSE

## Abstract

Each individual has a unique and interacting set of genetic, lifestyle, and environmental factors that are reflected in their physical exam and laboratory biomarkers and significantly impact their experience of health. Patterns of nutrient deficiency signs and biomarker levels below health-promoting thresholds have been identified in national nutrition surveys. However, identifying these patterns remains a challenge in clinical medicine for many reasons, including clinician training and education, clinical time restraints, and the belief that these signs are both rare and recognizable only in cases of severe nutritional deficiencies. With an increased interest in prevention and limited resources for comprehensive diagnostic evaluations, a functional nutrition evaluation may augment patient-centered screening evaluations and personalized wellness programs. During LIFEHOUSE, we have documented physical exam, anthropometric, and biomarker findings that may increase the recognition of these wellness-challenging patterns in a population of 369 adult employees working in two occupational areas: administrative/sales and manufacturing/warehouse. Distinct and significant physical exam differences and constellations of biomarker abnormalities were identified. We present these patterns of physical exam findings, anthropometrics, and advanced biomarkers to assist clinicians in diagnostic and therapeutic interventions that may stem the loss of function that precedes the development of the non-communicable chronic diseases of aging.

## 1. Introduction

Each individual has a unique and complex set of genetic, lifestyle, and environmental factors that are reflected in their physical exam and laboratory biomarkers, create potential disease burdens, and impact their health. In clinical practice, the identification of these examination and anthropometric findings and laboratory biomarkers may illuminate the preclinical mileposts of disease and illness that foretell the development of the common non-communicable chronic diseases (NCDs) of aging including heart disease [1,2], hypertension [3], type II diabetes mellitus [4], renal disease [5], and lung disease [6]. Although it was previously assumed that being overweight and/or obese contributed to the development of NCDs, it is now recognized that these changes in body composition may also be early signs of the pathophysiologic disruption associated with caloric excess, toxic burden [7], and changes in the gut microbiome [8]. Hence, a systems-based approach to quantifying wellness and detecting transitions to disease is well suited for preventing NCDs common to modernized societies [9].

Hood [10] suggested that the best medicine should value four principles; thus, P4 medicine is personalized, predictive, preventive, and participatory. For medicine to be predictive and preventative, clinicians need to identify early pathophysiologic changes associated with NCDs. However, identifying these patterns/changes remains a challenge in clinical medicine. There are many reasons for this, including clinician training and education, clinical time restraints, and the belief that nutrition insufficiency/deficiency signs are both rare and recognizable only in cases of severe nutritional deficiencies including beriberi, pellagra, scurvy, rickets, marasmus, or kwashiorkor. Although these classic nutritional-deficiency diseases are being diagnosed with decreasing frequency, Reber et al. report that the occurrence of nutritional insufficiency and deficiency is still missed frequently enough by clinicians that comprehensive nutritional screening and assessment methods can contribute to an effective and well-structured nutritional management (process cascade) for hospitalized patients [11].

Changing practice styles have deemphasized the physical exam in favor of evaluating an ever-expanding list of high-tech studies including laboratory biomarkers and imaging studies to diagnose the advanced stages of NCDs [12,13]. However, functional medicine and personalized lifestyle medicine have long recognized that ‘healthy’ individuals may present with recognizable patterns of pre-clinical disease associated with underlying nutritional insufficiencies/deficiencies related to genomic uniqueness, toxic exposures, and maladaptive lifestyle choices [14]. Marcucci Leao and dos Santos note that micronutrient intake deficiency is a global health problem affecting about 2 billion people and seems to be associated with an increased risk for non-communicable diseases and disorders, including obesity [15].

Gravallese and Firestein have demonstrated the utility of this model in a recent review of rheumatoid arthritis [16]. Ames’ “triage theory” proposes a causal link between the chronic, modest deficiency of a micronutrient and NCDs [17]. Fenech [18] has reported that higher levels of many micronutrients may be necessary for various DNA maintenance reactions and that the current RDAs for some micronutrients may be inadequate to protect against genomic instability. Indeed, dietary changes and novel physical challenges may result in the creation of unique nutritional needs within hours and hence influence the interplay of environment and genome in phenotypic expression.

Nutrient deficiency signs and biomarker levels below health-promoting thresholds have been identified in national nutrition surveys. In 2017, Bird et al. [19] reported on the risk of deficiency in multiple concurrent micronutrients in children and adults in the United States identified in the NHANES evaluation of over 15,000 individuals. They noted thatthirty-one percent of the U.S. population was at risk of at least one vitamin deficiency or anemia, with 23%, 6.3%, and 1.7% of the U.S. population at risk of deficiency in 1, 2, or 3–5 vitamins, respectively. This is not too surprising, since individuals, both those considered healthy and those with identifiable conditions, frequently face many challenges in meeting their daily nutrient requirements. An example was reported by Hwang et al. [20], who noted that 32% of American adults were not consuming the daily requirement of magnesium dietary intake of 4.5 milligrams per kilogram per day. Bird et al. [19] report a significantly higher deficiency risk was seen in women (37%), non-Hispanic blacks (55%), individuals from low income households (40%), or without a high school diploma (42%), and underweight (42%) or obese individuals (39%). 

A deficiency risk was most common in women 19–50 years (41%), and pregnant or breastfeeding women (47%).

An evolving definition of health recognizes that health is directly related to the functional capacity of the individual. Progressive changes in functional health status can represent early warning signs of later disease. Functional capacity may be categorized into five assessment areas: metabolic, physical, cognitive, emotional, and behavioral [21,22]. The nutrition adequacy leverage point for health vs. early disease changes is unique for all humans and directly reflects their functional capacities. A functional nutrition evaluation (FiNE), including anthropometrics, biomarker evaluation, a clinical exam, and diet evaluation (the ABCDs of the Institute for Functional Medicine’s Functional Nutrition Evaluation) [23] is uniquely suited to identifying these patterns presaging functional deficits and health decline.

One goal of the Lifestyle Intervention and Functional Evaluation—a Health OUtcomes SurvEy (LIFEHOUSE) project is to identify patterns of dysfunction with FiNE assessments to facilitate meaningful personalized lifestyle interventions. We have documented physical exam, anthropometric, and biomarker patterns that clarify the value of individual components of a nutritional exam in a resource-challenged environment. These findings may enable clinicians to better tailor their diagnostic plans and subsequently intervene with personalized modifiable lifestyle and dietary choices that impact health and modulate disease in healthy adults.

## 2. Methods

A full description of the rationale and methods of LIFEHOUSE has been reported [21]. The protocol for LIFEHOUSE, a survey ‘N-of-1’ trial featuring a tent, umbrella, and bucket design, has been reviewed and approved by the Aspire Independent Review Board (IRB) (Santee, CA, USA), now a member of the Western-Copernicus Group IRB. Our survey has been registered with ClinicalTrials.gov (accessed on 13 January 2022) as NCT04005456. Informed Consent was obtained from all participants prior to enrollment.

Approximately 400 male and female employees of a nutritional supplement company were recruited for participation in LIFEHOUSE. At regular intervals, participants were reassessed using validated functional assessment tools and a variety of biometric and laboratory evaluations. In this paper, we analyze the data of 369 participants who had met the minimal requirements for data analysis, which was deemed to consist of an initial clinical visit and baseline anthropometrics and serum biomarkers. As individuals had the option to decline specific assessments, some findings and biomarkers are noted to have a relatively smaller n. This group included adults between the ages of 18 and 64 and was composed of approximately sixty-five percent females and thirty-four percent males. Participants were employed as administrative staff (including company leadership), sales representatives, and manufacturing/warehouse personnel in five different locations (Aliso Viejo, CA, USA; Sante Fe Springs, CA, USA; Lenexa, KS, USA; Colonial Height, VA, USA; and Gig Harbor, WA, USA).

### 2.1. Physical Exam

A full description of the FiNE physical exam conducted during LIFEHOUSE, specifically the exam of the hair, mouth, skin, nail, peripheral nerve, smell, and bitter taste are detailed in Table 1. The FiNE exam focuses on readily accessible clinical exam components that reflect changes in health, disease, and nutrition adequacy. The FiNE was adapted and abbreviated from the Institute for Functional Medicine Functional ABCD, ref. [23] including the 8-step mouth exam [24]. Examinations and data/specimen collection were completed onsite for participants in their normal work routines. Our clinical team of six clinicians (Ph.D. nutritionists, naturopaths, nurse practitioners, and traditional MDs) performed the examinations. If there were uncertain findings, consultations within the group of clinicians were obtained for confirmation.

Smell was screened one nostril at a time after assuring that the nasal passage was patent. The UPSIT (smell identification test) 4 odor screening card from Sensonics International (Haddon Heights, NJ 08035, USA) was used to screen for anosmia, hyposmia, or normal smell. Bitter taste was screened with the PTC (phenylthiocarbamide) taste test from Precision Laboratories (Cottonwood, AZ 86326, USA). After first inquiring to make sure there was no known chemical sensitivity to the propylthiouracil or other chemical constituents, the PTC paper strip was placed on the anterior 1/3rd of the tongue, the saliva saturated the strip contacting the tongue and the participant reported whether the taste was strongly bitter, slightly bitter, or neutral.

As a component of the neurological exam, vibratory sense was assessed using a 128 Hz tuning fork to test sensitivity in the thumb and fifth digit of both upper extremities and the great toe and fifth digit of both lower extremities. While the examiners finger was placed under the digit being interrogated, the tuning fork was placed on the top of the digit distal to the distal interphalangeal joint. If there was no sensation, then the examiner would move proximally until the patient felt some vibration. A finding of decreased vibratory sense was recorded if abnormal at the first test site. Light touch was assessed, using medical grade 5.07/10 g monofilament (Medical Monofilament, Plymouth, MA 02360, USA) on the distal pads of the fingers and toes, specifically the thumb, first digit, and fifth digit of each hand and the great toe and fifth digit of each foot. If monofilament sensation was absent or not appreciated after a one-second hold and appropriate bending of the monofilament, this was recorded as negative sensation. The timed up and go test (TUG) was performed. While in a sitting position with their feet flat on the floor, they were asked to stand without using their arms, walk 3 m, turn around and walk back and sit down. If the timed up and go was less than 10 s, it was considered normal. If the time required was greater than 10 s, it was considered abnormal [25].

### 2.2. Anthropometrics

Anthropometric measurements included body temperature, blood pressure, pulse, respiratory rate, and oxygen saturation. Height, weight, waist circumference, hip circumference, body composition analysis, peak flow, and grip strength were measured. Body mass index (BMI) and waist-to-hip ratio were calculated. Body temperature was collected using a digital ear thermometer. Blood pressure and pulse were collected using appropriately sized cuffs and a Welch Allyn automatic 300-series sphygmomanometer (Skaneateles Falls, NY 13153, USA), with the participant sitting for 5 min with their non-dominant arm resting at heart level. Respirations were counted for 15 s, with the result multiplied by 4 and recorded as the respiratory rate. Oxygen saturation was recorded FL-100 Fingertip Pulse Oximeter (PulseOximeter, Buffalo Grove, IL 60089, USA) and recorded to the nearest percentage. Height was determined with a stadiometer recording height in inches to the quarter inch. The weight was documented with a standard digital scale of 0.1 lb. A Tanita bioelectrical impedance scale (Tanita Inner Scan BC-1000 Plus, Tanita Corporation of America, Arlington Heights, IL 60005, USA) was used to measure weight and assess body composition. Waist and hip measurements were completed with cloth tape in standard fashion. The World Health Organization published its report on waist circumference and waist-to-hip ratio, establishing standards, including the need to ensure accurate tape positioning, the use of proper tape, and the need for a constant 100 g of tension [26]. BMI was calculated in standard fashion (weight (kg)/height (m^2^)), with participants classified as underweight, desirable weight, overweight, or obese. For individuals in the overweight and obese categories: if the waist–hip ratio was greater than 0.80 for women and 0.85 for men, they were classified as having an android body type and if the waist-to-hip ratio was less than 0.80 for women and 0.85 for men, then they were classified as having a gynoid body type. Individuals with percentage body fat greater than 25% for men and 30% for women were considered overfat, even if the BMI category was underweight or desirable weight. Body composition (skeletal muscle mass and extracellular water/total body water calculations) offered the opportunity to further delineate metabolic health [27]. Peak flow was recorded using standard age- and sex-adjusted norm using a Respironics, peak flow meter (Phillips USA, Cambridge, MA 02141, USA). Three readings were obtained, and the average of the readings was calculated and recorded. Grip strength was determined using a Jamar hydraulic hand dynamometer (Sammons Preston, Saint Paul, MN 55101, USA) in both the dominant and nondominant hands. Standard NHANES gender-specific and age-specific ranges were used to assess grip strength and recorded.

### 2.3. Laboratory Biomarkers

Laboratory serum/plasma biomarkers were tested by Cleveland Heart Lab (6701 Carnegie Ave, Suite 500, Cleveland, OH 44103, USA) and its national reference laboratory partner, Quest Diagnostics (1737 Airport Way S, Seattle, WA 98134, USA). Stool was collected and a microbiome assessment was conducted (Genova Diagnostics, 63 Zillicoa Street, Asheville, NC 28801, USA).

### 2.4. Statistical Analysis

From the LIFEHOUSE data set, we selected the data of 369 participants who had met the minimal requirements for data analysis that was deemed to consist of an initial clinical visit and baseline anthropometrics and serum biomarkers. Missing data were not imputed. Standard estimation techniques, both paired and unpaired *t*-tests, were conducted as appropriate. As applicable, non-parametric tests, including the chi-square test and Fisher’s exact test, were conducted. For simplicity, significance is reported as nonsignificant (NS, >0.05) or significant (≤0.05, ≤0.01, and ≤0.001). Primary data analysis was conducted by Bennett Data Sciences (San Diego, CA 92107, USA).

## 3. Results

### 3.1. Description of Population

The age and sex distribution (Table 2), baseline characteristics of age, body mass index, waist circumference, and ethnicity (Table 3), and body composition by sex, age, and occupational function (Table 4) demonstrate the broad range of participants and the uniqueness of the population. Males have a higher BMI, larger waist circumferences, and a greater percentage of android obesity. This is consistent with the American population for adults in this age range [28] and is paradoxically influenced by gender, socioeconomic pressures, and food security [29]. When comparing the body composition by sex, age, and occupation (Table 4), there is a significant minority of females and a very few males that are gynoid obese, characterized by a BMI > 25.0 without an elevated waist-to-hip ratio. Females had a significantly higher percentage of normal BMI (≥18.5 and <25.0) (*p* ≤ 0.01) but elevated fat percentage (overfat) (*p* ≤ 0.01) then the males. Waist circumference (*p* ≤ 0.05) and body fat (*p* ≤ 0.01) increased significantly with age, with 67% of those older than 35 years of age having abnormally high body fat (>30% body fat for females and >25% body fat for males).

Occupational roles were associated with significant differences in body composition. Administrative/sales participants had a significantly decreased frequency of increased BMI (52% vs. 66%, *p* ≤ 0.05). In addition to an increased BMI, manufacturing/warehouse participants had increased waist circumference (59% vs. 30%, *p* ≤ 0.001), increased waist-to-hip ratio (52% vs. 66%, *p* ≤ 0.05), and high body fat (74% vs. 57%, *p* ≤ 0.01). Both populations had relatively few participants with a low BMI and though non-significant, the incidence was greater in the manufacturing/warehouse population. Over half the participants with low BMI were noted to have an associated history of persistent inflammatory gastrointestinal conditions.

### 3.2. Exam, Anthropometric, and Biomarker Observations

Males had a greater prevalence for hypertension (36%) than females (18%) (*p* ≤ 0.001) and it was skewed by age and work function (Supplemental Table S1). In males over 35 years of age, 25% had documented non-symptomatic hypertension. The manufacturing/warehouse participants had a 33% incidence of hypertension. Women did not have significant differences in hypertension associated with age or occupation. Males have a higher incidence of documented hypertension in the American population than females, and blood pressure is influenced by urban, small city, or rural settings [30,31]. Participants in our study generally lived in urban/suburban environments. In this population, occupation was associated with increased hypertension, with those in manufacturing/warehouse roles having significantly more hypertension compared with those administrative/sales roles (37% vs. 16%, *p* ≤ 0.001). Older participants were also noted to have an increased incidence of hypertension (27% vs. 13%, *p* ≤ 0.05).

The FiNE demonstrated many significant differences between males and females, by age, and by occupation in physical exam findings (Supplemental Table S2). There were sex differences in the incidence of hair loss, with males having 43% balding and females having 7% androgenic alopecia (*p* ≤ 0.001), with androgenic alopecia in women only seen in women over the age of 35 (*p* ≤ 0.05).

The nail growth patterns with longitudinal or vertical ridging were present in 27% and 29% of males and females. The nail shape was deemed abnormal on exam more often with age and was more common in those who worked in manufacturing/warehouse. Interestingly, nails were more brittle and weaker in the administrative/sales population than in the manufacturing/warehouse population. The degree of brittleness was not significantly different with age. As expected, females had more artificial nails than males. No one had artificial nails working in manufacturing/warehouse.

No significant differences in incidence of skin diseases were noted between the administrative/sales population and the manufacturing/warehouse population. There was a trend towards significance for the incidence of seborrhea, which was more common in the administrative/sales population (*p* = 0.06). There were no significant differences by age, sex, or occupation in the other skin findings of xerosis, dryness, and follicular hyperkeratosis. The female incidence of eczematous rash and hyperkeratosis pilaris was 3.5%, with the male incidence at 4.7%. Those individuals with eczema had increased omega-6 levels (NS) and associated alterations in nail growth patterns (ridging—longitudinal or transverse) (*p* ≤ 0.01) but without the findings commonly associated with psoriasis. Those individuals with seborrhea had significantly increased total omega-6 levels (*p* ≤ 0.05), increased tissue transglutaminase IgG levels (*p* ≤ 0.01), and tended to also have increased tongue coating on physical exam (*p* ≤ 0.05). Individuals with xerosis had significantly increased omega-3 fatty acids (*p* ≤ 0.05), increased 25 hydroxy vitamin D (*p* ≤ 0.05), and increased homocysteine (*p* ≤ 0.01).

Our oral exam findings are summarized in Supplemental Table S3. In this population, males have significantly less healthy gums than females (*p* ≤ 0.01), with 34% of males and 16% of females having obvious inflammation of the gums. Males had significantly more periodontal disease than females (8% vs. 2%, *p* ≤ 0.05). Over 30% of both males and females had either a white or yellow non-adherent tongue coating. Males had significantly more yellow coating than females (*p* ≤ 0.05). Tongue coating was associated with a significantly higher incidence of food IgG hypersensitivity (*p* ≤ 0.05) and IgA and IgG gliadin (*p* ≤ 0.020580205 and NS respectively). Tongue coating was associated with an increased percentage of body fat (*p* ≤ 0.05). Although the exam was not a formal dental exam but rather a simple screening exam noting missing teeth, whether there were restorations, or whether there was untreated dental caries, this pattern is suggestive of oral dysbiosis and systemic inflammatory conditions. These findings were associated with increased hs-CRP (*p* ≤ 0.05), HgbA1c (*p* ≤ 0.01), HOMA-IR (*p* ≤ 0.05), BMI (*p* ≤ 0.05), elevated blood pressure (*p* ≤ 0.05), decreased vibratory sense (*p* ≤ 0.05), and monofilament-associated neuropathy (*p* ≤ 0.05). The older workers had more dental abnormalities (68% vs. 41%, *p* ≤ 0.001). The manufacturing/warehouse participants had markedly worse dental health than the administrative/sales population with more dental abnormalities (77% vs. 53%, *p* ≤ 0.001) and more untreated dental caries (18% vs. 3%, *p* ≤ 0.001).

Change in taste or smell affects food choice—loss of taste and smell is associated with increased salt, sugar, and fat consumption [32]. Females presented more frequently with decreased smell than males (11% vs. 4%, NS). More females were bitter taste super tasters (24% vs. 13%, *p* ≤ 0.01). Bitter taste receptors, found in many extra-oral sites, are involved in the innate immunity and metabolic function [33]. We found that decreased bitter taste perception was significantly positively associated (*p* ≤ 0.05) with HbA1c, IgA gliadin, IgG gliadin, increased fecal akkermansia, and blood pressure and significantly negatively associated (*p* ≤ 0.05) with 25-OH vitamin D.

We found that older participants had increasing difficulty with single leg balance (closed eyes; left leg balance: 68% vs. 87%, *p* ≤ 0.01) (closed eyes; right leg balance: 74% vs. 88%, *p* ≤ 0.05). Males had decreased light touch in the LE compared with females (91% vs. 97%, *p* ≤ 0.05). Older participants had decreased vibratory sense in both lower (45% vs. 24%, *p* ≤ 0.05) and upper extremities (14% vs. 5%, *p* ≤ 0.05). Manufacturing/warehouse vs. administrative/sales experience had decreased vibratory sense in both lower (65% vs. 28%, *p* ≤ 0.01) and upper extremities (23% vs. 7%, *p* ≤ 0.001).

### 3.3. Pattern Recognition Observations

Recognizing the basic principles of functional medicine (biochemical individuality; the dynamic balance among internal and external factors reflected in modifiable lifestyle behaviors; the web-like connections of eight areas of physiologic balance; health as positive vitality and function and not the simple absence of disease) provides a heuristic for early recognition of pre-clinical patterns that precede the development of symptomatic NCDs [34]. Although many of these patterns are currently substantiated in the literature, some are less strongly clinically validated. The authors identified a group of associations for which statistical confirmation of known associations and validation of hypothesized associations were sought. Attention is directed only to positive findings as the number available for analysis may have been too small to exclude false-negative associations.

Table 5 (Statistically Significant Associations between Physical Exam/Anthropometric Findings and Biomarkers) and Table 6 (Statistically Significant Associations between Biomarkers and Physical Exam/Anthropometric Findings) present our early findings of associations with statistical significance (*p* values ≤ 0.05). Ongoing principal component analyses are now being conducted to look for novel findings that maybe hidden from clinical eyes but discoverable with advanced statistical methods.

We noted significant expected associations between normal BMI vs. overweight and obese BMI and metabolic markers (HOMA-IR, HbA1c, hs-CRP, triglycerides, LDL particle number, elevated blood pressure). Paradoxically, being overweight was associated with a decreased firmicutes/bacteroidetes ratio and not associated with Akkermansia levels. Interestingly, being overweight or obese is significantly associated with decreased 25-OH vitamin D levels.

Skin findings were frequently significantly associated with levels of the essential fatty acids. Seborrhea was positively associated with omega-6 fatty acids levels, xerosis was positively associated with omega-3 fatty acids level, and both hyperkeratosis pilaris and acne vulgaris were negatively associated with omega-3 and omega-6 levels. Lip cracking was also positively significantly associated with omega-6 levels and negatively associated with omega-3 levels.

Markers of food hypersensitivity were significantly associated with tongue findings—both coating and fissuring as well as skin findings including seborrhea, hyperkeratosis pilaris, and acne vulgaris. Abnormal taste and smell perception and abnormal balance and grip strength were all negatively associated with 25-OH vitamin D levels.

## 4. Discussion

In our ongoing review of the extensive survey database (>100 K individual data points not inclusive of genomic data), we have confirmed findings previously observed including an increase in abnormal findings with increased age, gender differences (obesity and hypertension in particular), and differences between occupations likely due to the influence of socio-economic status and challenges.

### 4.1. Body Compositions

Half of our participants with a low BMI (<18.5 kg/m^2^) had a history of persistent inflammatory gastrointestinal conditions. Chronic inflammatory bowel conditions are associated with an increased incidence of nutrition insufficiency and abnormal biomarkers in over 20% of patients seen in the outpatient clinic setting [20] and clinical malnutrition in 16 percent of IBD patients [35]. Uncovering nutritional insufficiencies for these participants can help in the development of a personalized response to the functional deficits and improve their health [36].

Our findings support the broader role different types of obesity may play in human health and disease. Gynoid obese individuals more often have associated endocrinologic conditions (hypothyroidism with elevated TSH, low T3, T4), androgen disruption with low estradiol, or toxicity with elevated RBC heavy metals, bis-phenyl A, or the metabolic byproduct of small intestinal bacterial overgrowth, d-lactate [37]. Individuals with normal BMI but elevated waist-to-hip ratios are often metabolically less healthy. It is known that adipocytes in different regions have different inflammatory activities and metabolic characteristics, with visceral obesity being frequently characterized as ‘angry’ fat. In women with a low waist-to-hip ratio and lower body obesity vs. women with an android high waist-to-hip ratio and upper body obesity, there is differential adipocyte activity. Abdominal adipocytes tend to enlarge, whereas subcutaneous femoral adipocytes increase in number [38]. In fact, there are increased adipogenic transcription factors (CCAT/enhancer-binding protein alpha and PPAR gamma 2) in hypertrophic (enlarging) adipocytes [39]. Indeed, hypertrophic adipocytes tend to be more associated with insulin resistance and dyslipidemia [40]. Additionally, it appears that the gluteal fat deposits correspond more closely with visceral deposits rather than femoral deposits in their activity [41,42]. From a biomarker indicator, subcutaneous fat is a major contributor to systemic circulating free fatty acids. It contributes more to serum levels than visceral or retroperitoneal fat and plays a role in insulin resistance [43]. No matter where the fat is distributed, adipose tissue macrophage infiltration is associated with the elevation in hs-CRP levels [44]. The health of the adipose tissue and its role in driving inflammation and insulin resistance is associated with other markers, including IL-1, IL-6, and TNF-alpha [45]. Bays [46] has suggested that ‘sick fat’, or adiposopathy, causes high blood sugar, high blood pressure, and dyslipidemia.

We noted significant expected associations between BMI and metabolic markers (HbA1c, hs-CRP, triglycerides, LDL particle number, blood pressure, and Firmicutes/Bacteroidetes ratio). It is interesting to note that being overweight or obese is significantly associated with decreased 25-OH vitamin D levels. Abnormal taste and smell perception and abnormal balance and grip strength were all negatively associated with 25-OH vitamin D levels. Recently, it has been suggested by Cummings and Rosen that it is not advised to measure vitamin D levels in generally ‘healthy’ adults [47]. Although this conclusion has generated much controversy, these patterns may help justify a clinician’s desire to measure 25-OH vitamin D levels or supplement with vitamin D3.

### 4.2. Nail and Skin Findings

We found that greater than 25% of participants had vertical ridging of their fingernails. Whereas vertical ridging can be a normal variant, it is also associated with maldigestion or malabsorption, autoimmune conditions such as rheumatoid arthritis and psoriasis, and peripheral circulatory inadequacy [48,49,50]. Maldigestion or malabsorption due to prolonged use of gastric acid modulators is a common occurrence in the population in this age group [51]. Observing vertical ridging is an opportunity to correlate this finding with other physical exam and biomarker findings; when nail ridging is associated with malabsorption of protein or fats, the patient will present with decreased muscle mass, increased skin dryness, or even peripheral neuropathy.

We noted a less than expected incidence of eczematous rash and hyperkeratosis pilaris. This may be influenced by the relatively optimal omega-3 serum levels in this population of participants with access to regular omega-3 supplementation. We noted that participants with seborrhea had statistically significantly elevated levels of omega -6 fatty acids. Our findings confirm the prior association between both eczema and hyperkeratosis pilaris incidence and dietary and biochemical adequacy of long-chain essential fatty acids and a constellation of other nutrients [52].

A skin exam documenting seborrhea, xerosis, hyperkeratosis pilaris, and acne vulgaris, as well as the finding of lip cracking, may provide the impetus to measure omega-3 and omega-6 levels. We noted that participants with xerosis had significantly increased omega-3 fatty acids, increased 25 hydroxy vitamin D (*p* ≤ 0.05), and increased homocysteine (NS). Xerosis is often seen in individuals with type 2 DM or peripheral vascular disease, yet in this population there was no association with elevated glucose, HbA1c, insulin, or HOMA-IR. These associations in physical exam findings may be influenced by the many interactions between body weight, inflammatory imbalances from inadequate lipid modulators in the NCD setting, and even the gut microbiota. There is an association between obesity and psoriasis [53], the microbiota and psoriasis [54], and metabolic syndrome and psoriasis [55].

### 4.3. Oral Exam

The mouth exam is often overlooked during the routine clinical exams. Over 33% of adults in the United States do not have access to dental care [56]. It is acknowledged that there is a significant connection between oral health and systemic disease [57,58]. Oral dysbiosis is associated with an increased tongue coating [59]. A tongue coating may be suggestive of a more inflammatory oral microbiome. Oral dysbiosis can be associated with an increased hs-CRP and lower vitamin D levels and increased immunologic reactions to gluten [60]. We found that tongue coating was associated with a higher incidence of food IgG-based hypersensitivity and elevated IgA/IgG gliadin antibodies.

Missing teeth, extensive dental restorations, and untreated dental caries is suggestive of oral dysbiosis and systemic inflammatory conditions. Our survey confirms this hypothesis by finding an association between these collective findings and increased hs-CRP, increased insulin levels, increased HOMA-IR and HbA1c, elevated BP, and peripheral sensory exam changes.

Markers of food hypersensitivity were significantly associated with tongue findings—both coating and fissuring as well as skin findings including seborrhea, hyperkeratosis pilaris, and acne vulgaris. The clinical symptoms of food hypersensitivity are frequently vague and can be rather diverse. Seeking these physical exam findings may increase the confidence with which testing for gluten hypersensitivity or food allergies is sought or for prescribing a modified food elimination program.

### 4.4. Strengths and Limitations

Our population is an employee group working for a nutritional supplement company with a broad interest in promoting health. These participants regularly receive invitations to participate in health interventions and indeed participated in our study. Despite this focus and despite some indications of better nutritional status (omega-3 fatty acid levels), our participants reflect the influences of our current socio-economic environments and are indeed reflective of the broader population. Strengths include the broad range of laboratory biomarkers and extensive anthropometric and physical exam markers collected on each participant, as well as the unique adaptive ‘N-of-1’ tent, umbrella, and bucket design that met each participant with a personalized approach. Limitations include the relatively small number of participants, the limited ethnic diversity, and the fact that this represents an adult population. Planning is currently underway for LIFEHOUSE 2.0, which will expand our survey into the primary care setting.

## 5. Conclusions

By necessity, the national healthcare model has adopted a primarily disease-care focus due to changing demographics and increasing limits on economic resources. Indeed, estimates suggest that 70–80% of resources are directed towards providing acute care responses to NCDs, with clearly limited benefit. To facilitate increased opportunities for addressing loss of function during the often silent pre-clinical phase of pathophysiological changes resulting in ICD-10 definable disease states, we need new tools. The FiNE, with its ability to identify patterns of early pathophysiology and the impending loss of function, is one of these tools. Our clinical team, consisting of medical assistants, Ph.D. nutritionists, naturopaths, nurse practitioners, and traditional MDs, highlights that many portions of the FiNE can be conducted by members of the care team other than solely physicians. Hence, the FiNE may potentially be of use in the busy office setting during annual wellness exams, in employee health settings, in community-based health screening programs, and in medically indigent societies and communities.

Although confirmatory of prior work, our data regarding body composition with the associated changes in inflammatory markers, support the broadening recognition of the importance of lifestyle choices in early loss of function and development of disease. Pathophysiological changes seen in many NCDs (both condition related and those exacerbated by drug–nutrient interactions) can alter an individual’s unique nutrient needs [61]. Recognizing this type of pattern provides clinicians insight in crafting diagnostic plans—looking for proof of deficiency states and early biomarkers of disease—as well as developing personalized nutritional recommendations targeted to a person’s unique phenotype and needs. Currently, members of our group are involved with the development of tools that will facilitate the collection and collation of these observations for use by busy clinicians.

We believe the evidence presented here regarding pattern recognition of early pathophysiologic changes associated with loss of function and the development of pre-clinical dysfunction may have far reaching impact in reducing the burden of NCDs that affect so many individuals. Clearly, more work with a larger population needs to be undertaken to confirm our conclusions; however, in the interim, it is hoped that the identified patterns may assist clinicians and their patients in choosing practical lifestyle interventions.

## Figures and Tables

**Table 1:** Components of the FiNE; Anthropometric and Clinical Exam.

**Anthropometrics**	Body temperature, blood pressure, pulse, respiratory rate and oxygen saturation. Height, weight, waist circumference, hip circumference, body composition analysis, peak flow, and grip strength
	Body Composition Evaluation: Body Mass Index, waist to hip ratio, percent body fat, skeletal muscle mass, extracellular water/total body water
	Body Type: Underweight, desirable weight, overweight, obese, over fat, android body type, gynoid body type
**Clinical Exam**	
**Scalp Hair**	Distribution: Normal, alopecia areata, androgenic alopecia (female)
**Mouth**	Jaw Movement: symmetric, asymmetric, auscultated crepitus or click, pain, mouth opening >4 cm. Lips: Normal, dry, cracking, angular cracks or sores, ulcerations, fissures, perioral rash, loss of lip borders, other lesions, edema, angioedema, piercings. Soft palate, hard palate, tonsillar pillars: hard palate—normal, cleft or oropharyngeal defects. Soft palate—lesions, symmetry. Tonsil beds and pillars—hypertrophy, regressed. Tongue: size-enlarged, small, shape-scalloping present, color- red, magenta, coating- grade 0, 1, 2 and color, tastebud distribution and prominence, fissuring (longitudinal, transverse, lambda) ankyloglossia, lesions, ventral varicosities, blocked Wharton duct blocked. Gums: normal, lesions, gum line darkening-burtons lines, bruising, lesions, macules, tenderness, gingivitis, periodontal disease, gum hyperplasia. Teeth: Healthy-no restorations, missing teeth, tooth attrition or abrasions, silver/mercury restorations, silver abutting gold restorations, periodontal ligament pain, enamel dysplasia, discoloration (fluorosis) plaque (tartar).
**Skin**	Texture: normal, xerosis, hyperkeratosis pilari, seborrhea, eczematous rash. Color: Normal, acanthosis nigricans, ecchymosis Hair: distribution, swan neck hairs Lesions: normal, acne vulgaris, keratosis (seborrheic, actinic, arsenic), cancers: basal cell, squamous cell, melanoma, poor wound healing.
**Fingernails, Toenails**	Shape: normal, koilonychia, parrot Color: normal, terry nails, leukonychia Texture: brittle, chipping nails Growth pattern changes: Beau’s lines, longitudinal ridging, nail beading Artificial surfaces: acrylic nails, or polished nails
**Neurological exam**	monofilament (5.07/10 g) vibratory sense (128 Hz) balance standing (eyes closed) single leg stand (eyes closed) motor- Timed Up and Go smell test (cranial nerve 1) taste test (bitter) (cranial nerves, 7, 9, 10)

**Table 2:** Study Population; Sex and Age Range Distribution.

Characteristic	Male; Percent (n)	Female; Percent (n)
Sex	34.42 (127)	65.58% (242)
Age, average (years)	41.02	43.03
18–25	4.72 (6)	4.96 (12)
26–35	31.5 (40)	22.73 (55)
36–45	32.28 (41)	30.58 (74)
46–65	18.9 (24)	26.45 (64)

**Table 3:** Baseline Characteristics of Participants of the LIFEHOUSE Design Survey; Age, Body Mass Index (BMI), Waist Circumference, Ethnicity.

Descriptive	Total (n = 369)	Admin/Sales (n = 257)	Manufacturing (n = 112)
Age (years)	42.3 ± 10.9	42.8 ± 10.1	41.4 ± 12.5
BMI (kg/m^2^)	27.4 ± 6.0	26.5 ± 5.1	30.5 ± 7.4 *
BMI (kg/m^2^) female	26.5 ± 6.0	25.7 ± 5.4	29.5 ± 7.0 *
BMI (kg/m^2^) male	29.4 ± 5.7	28.4 ± 4.1	31.7 ± 7.8 *
Waist Circumference (cm)	91.4 ± 15.5	88.6 ± 14.0	99.3 ± 16.8 *
Waist Circumference (cm) Female	86.6 ± 14.2	84.1 ± 13.0	94.0 ± 15.5 *
Waist circumference (cm) Male	100.1 ± 14.0	94.5 ± 12	108.2 ± 15.0 *
Sex (% female)	242 (65.6%)	175 (68.1%)	67 (59.8%)
Ethnicity/race			
African American	14 (6.2%)	9 (5%)	5 (10.9%)
Asian	25 (11%)	21 (11.6%)	4 (8.7%)
White	147 (64.8%)	120 (66.3%)	27 (58.7%)
Hispanic	27 (11.9%)	17 (9.4%)	10 (21.7%)
Native American	8 (3.5%)	8 (4.4%)	
Other	6 (2.6%)	6 (3.3)	

* Indicates significance for higher body mass index (BMI) (kg/m^2^) when compared to Corporate/Sales (*p* < 0.05).

**Table 4:** Body Composition by Sex, Age, Occupation.

Body Composition			
*Sex Comparison*	Male, % (n)	Female, % (n)	*p* Value
Normal	30 (29)	50 (93)	≤0.01
Elevated BMI	72 (71)	47 (88)	≤0.001
Elevated Waist Circumference	48 (47)	34 (63)	≤0.05
***Body Type***			
Android	60 (58)	41 (76)	≤0.01
Gynoid	1 (1)	12 (23)	≤0.01
Over Fat	3 (3)	9 (18)	NS
***Age Comparison***	**Age 18–35, % (n)**	**>35 %, (n)**	***p* value**
Elevated WC	29 (24)	43 (86)	≤0.05
Abnormal body fat	48 (40)	67 (136)	≤0.01
***Occupation Comparison***	**Admin/Sales, % (n)**	**Manufacturing, % (n)**	***p* value**
Normal	48 (96)	32 (26)	≤0.05
Elevated BMI	52 (105)	66 (54)	≤0.05
Low BMI	0 (1)	4 (3)	NS
Elevated WC	30 (62)	59 (48)	≤0.001
Elevated WHR	47 (95)	60 (49)	NS
Abnormal body fat	57 (115)	74 (61)	≤0.01
***Body Type***			
Android	41 (83)	63 (51)	≤0.01
Gynoid	18 (8)	6 (7)	NS
Normal	45 (91)	27 (22)	≤0.01

Abbreviations/Explanations: Elevated BMI > 25.0; Elevated waist circumference > 36″ female, >40″ male; Low BMI < 18.5; Android body type—waist to hip ratio > 0.80 female, >0.90 male with elevated BMI and body fat percentage; Gynoid body type—waist to hip ratio < 0.80 female, <0.90 male with elevated BMI, and body fat percentage; Over Fat—BMI < 25.0 with elevated body fat percentage; Elevated waist to hip ratio > 0.80 female, >0.90 male; Abnormal Body Fat: >30% female, >20% male.

**Table 5:** Statistically Significant Associations (*p* ≤ 0.05) between Physical Exam/Anthropometric Findings and BioMarkers.

Anthropometrics	Significant Biomarker Patterns
**Body composition**	
BMI < 30.0 v. ≥30.0	BMI ≥ 30.0: increased HbA1c, hs-CRP, TG, LDL particle number (LDLp), and BP; decreased vitamin D, Firmicutes/Bacteroidetes Ratio
BMI < 25.0 v. ≥25.0	BMI ≥ 25.0: increased HOMA-IR, Hgb A1c, hs-CRP, TG, LDLp, BP; Decreased 25-OH Vitamin D, diminished monofilament sensation.
BMI < 25.0 v. ≥25.0, <30.0 v. ≥30.0	Higher the BMI: increased HOMA-IR, hemoglobin A1c, hs-CRP, TG, LDLp, BP; decreased 25-OH Vitamin D.
Elevated Waist Circumference	Increased HOMA-IR, HbA1c, hs-CRP, TG, LDLp, Increased BP, Decreased 25-OH Vitamin D, Firmicutes/ Bacteroidetes ratio
**Physical Exam**	
Skin Findings	Seborrhea: positive Omega 6, TTG IgG, and tongue coating
	Hyperkeratosis pilari: positive TTG IgA, TTG IgG, HbA1c, insulin, HOMA-IR, homocysteine, brittle nails; negative omega 6, omega 3
	Xerosis: positive omega 3, vitamin D, homocysteine
	Acne Vulgaris: positive TTG IgA, TTG IgG, decreased Firmicutes/Bacteroidetes, insulin, fecal sIgA, leukonychia; negative omega 3, omega 6, hyperkeratosis pilari.
Oral findings	Lip Cracking: positive omega 6, leukonychia, brittle nails; negative Omega 3
	Tongue Coating; positive IgG food allergies, Gliadin IgA, IgG; TG, LDLp, anti-CCP
	Tongue Fissuring: positive TTG IgG, gliadin IgA, IgG, BP, oral restorations, missing teeth; positive(unexpected) 25-OH Vitamin D, Firmicutes/Bacteroidetes ratio, increased fecal Akkermansia, hs-CRP, RF, anti- CCP, TPO, ANA
	Missing Teeth/Oral Restorations: positive HOMA-IR, HbA1c, hs-CRP, BMI, BP, decreased vibratory sense, abnormal monofilament test; negative 25-OH Vitamin D
Neurologic Exam	Decreased Bitter Taste: positive HbA1c, IgA gliadin, IgG gliadin, increased fecal akkermansia, BP; negative 25-OH Vitamin D
	Abnormal Smell Perception: positive TTG IgA, TTG IgG, increased fecal akkermansia, Firmicutes/Bacteroidetes ratio; negative 25-OH Vitamin D.
	Abnormal balance (One Leg): positive TG, % body fat, BMI, hsCRP, TPO; negative: 25-OH Vitamin D
	Abnormal Balance (Closed Eyes, Two legs): positive anti-gliadin IgA, increased TG, LDLp; negative 25-OH Vitamin D
	Grip Strength: Weak grip positive association–HOMA-IR; negative 25-OH Vitamin; negative TPO. U-Shaped curve noted for grip strength with BMI.

Abbreviations/Explanations: ANA—anti-nuclear antibody; Anti-CCP—anti-cyclic citrullinated peptide antibodies; BMI—Body Mass Index; BP—blood pressure; LDLp—LDL particle number; F/B ratio—Firmicutes/Bacteroidetes ratio; HbA1c—Hemoglobin A1c; HOMA-IR—homeostatic model assessment for insulin resistance; IgG—immune globulin G; RF—rheumatoid factor; sIgA—serum immune globulin A; TG—triglycerides; TPO—Thyroid peroxidase antibodies; TTG IgA—tissue transglutaminase immune globulin A; TTG IgG—tissue transglutaminase immune globulin G.

**Table 6:** Statistically Significant Associations (*p* ≤ 0.05) between Biomarkers and Physical Exam/Anthropometric Findings.

Biomarkers	Significant Exam Findings
hs-CRP	Positive waist circumference, BMI > 25.0, missing teeth/oral restorations, abnormal one leg balance
HOMA-IR	Positive waist circumference, BMI > 25.0, missing teeth/oral restorations
HgbA1c	Positive waist circumference, BMI > 25.0, missing teeth/oral restorations, no bitter taste
25-OH Vitamin D	Negative waist circumference, BMI > 25.0, no bitter taste, decreased smell, decreased grip strength
Homocysteine > 9.0	Positive Acanthosis nigricans, xerosis
Secretory IgA elevation	Positive eczema
TTG IgG	Positive seborrhea, tongue fissuring, decreased smell
Gliadin IgA, IgG	Positive tongue coating, tongue fissuring, no bitter taste, decreased smell
Triglycerides	Positive BMI > 25.0, waist circumference, decreased one leg balance, abnormal eyes closed two feet balance
LDLp	Positive: BMI > 25.0, waist circumference, abnormal eyes closed two feet balance
Omega 6 levels	Positive: seborrhea, eczema,
Omega 3 levels	Positive: lip cracking, xerosis,
Firmicutes/Bacteroides Ratio	Positive: eczema; negative: BMI > 25.0, >30.0, waist circumference, acne, abnormal smell perception
Elevated fecal Akkermansia	Positive no bitter taste, decreased smell

Abbreviations/Explanations: ANA—anti-nuclear antibody; Anti-CCP—anti-cyclic citrullinated peptide antibodies; BMI—Body Mass Index; BP—blood pressure; LDLp—LDL particle number; F/B ratio—Firmicutes/Bacteroidetes ratio; HbA1c—Hemoglobin A1c; HOMA-IR—homeostatic model assessment for insulin resistance; hs-CRP—high sensitivity C-reactive protein; IgA—Immune globulin A; IgG—immune globulin G; RF—rheumatoid factor; sIgA—serum immune globulin A; TG—triglycerides; TPO—Thyroid peroxidase antibodies; TTG IgA—tissue transglutaminase immune globulin A; TTG IgG—tissue transglutaminase immune globulin G.

## Data Availability

Data is contained within the article.

## Supplementary Materials

The following supporting information can be downloaded at: https://www.mdpi.com/article/10.3390/jpm13040594/s1, Table S1: Blood Pressure Comparison by Sex, Age, and Occupation. Table S2: Physical Exam Findings for Hair, Skin, and Nails Comparison by Sex, Age, and Occupation. Table S3: Physical Exam Findings for Mouth, Tongue, Teeth, Comparison by Sex, Age, and Occupation. Table S4: Physical Exam finding for Smell, Taste and Sensory Neurological Exam Comparison by Sex, Age, and Occupation.
