# Supplementary material for: LIFEHOUSE’s Functional Nutrition Examination (Physical Exam, Anthropometrics, and Selected Biomarkers) Informs Personalized Wellness Interventions
Source: J Pers Med. 2023 Mar 28;13(4):594. doi: 10.3390/jpm13040594 (PMC10145881; doi:10.3390/jpm13040594)
Supplement: Supplementary file 1 [file jpm-13-00594-s001.zip › jpm-2169037-supplementary.pdf]

Supplemental Tables

Supplemental Table S1: Blood Pressure Comparison by Sex, Age, and Occupation

**Blood Pressure**			
Sex Comparison	**Male, % (n)**	**Female, % (n)**	**p value**
Blood pressure - Hypertensive	35 (27)	17 (27)	0.01
Blood pressure - normal	59 (44)	71 (112)	NS
Age Comparison	**Age 18-35, %, (n)**	**>35, % (n)**	**p value**
Blood pressure - hypertensive	13 (8)	27 (45)	0.05
Occupation Comparison	**Admin/Sales, % (n)**	**Manufacturing, % (n)**	**p value**
Normal	70 (111)	61 (45)	0.01
Blood pressure - Hypertension	16 (26)	37 (27)	0.001

Supplemental Table S2: Physical Exam Findings for Hair, Skin, Nails Comparison by Sex, Age, and Occupation

PE Finding			
Sex Comparison	**Male, % (n)**	**Female, % (n)**	**p value**
**Hair**			
Alopecia	43 (41)	6 (12)	≤0.001
Androgenic alopecia	0 (0)	7 (14)	≤0.01
Normal	53 (51)	82 (153)	≤0.001
**Nails**			
Nail Polish/Artificial	3 (1)	40 (23)	≤0.01
Growth, longitudinal or vertical ridging	28 (27)	17 (29)	≤0.05
Age Comparison	**Age 18-35, % (n)**	**>35, % (n)**	**p value**
**Hair**			
Androgenic alopecia	0 (0)	7 (14)	≤0.05
**Nails**			
Normal shape	98 (78)	86 (166)	≤0.01
Occupation Comparison	**Admin/Sales, % (n)**	**Manufacturing, % (n)**	**p value**
**Nails**			
Brittle	12 (22)	2 (2)	≤0.05
Growth – Beau's lines	0 (0)	4 (3)	≤0.05
Growth patterns vertical ridging	27 (50)	8 (6)	≤0.001
Normal Nails	65 (121)	76 (61)	NS
Shape normal	92 (178)	81 (66)	≤0.05
Texture - brittle nails, weak, thin, bend easily	5 (10)	0 (0)	≤0.05
**Skin**			
Texture, seborrhea	4 (9)	0 (0)	NS

Supplemental Table S3. Physical Exam Findings for Mouth, Tongue, Teeth, Comparison by Sex, Age, and Occupation

**PE Finding**			
**Comparison by Sex**	**Male, % (n)**	**Female, % (n)**	**p value**
**Mouth**			
**Gums**			
Gums - normal	69 (66)	84 (157)	≤0.01
Gums - gingivitis	14 (13)	6 (12)	NS
Gums - periodontitis	8 (8)	2 (5)	≤0.05
**Tongue**			
Tongue fissuring - central	28 (26)	17 (32)	NS
Tongue shape	69 (65)	59 (110)	NS
Tongue scalloping	15 (9)	18.9 (21)	NS
Tongue coating - white or yellow	31.6 (19)	30.3 (34)	NS
**Teeth**			
Restorations	53 (50)	66 (123)	≤0.05
**Comparison by Age**	**Age 18-35, % (n)**	**>35, % (n)**	**p value**
**Gums**			
Gum line color abnormal	11 (9)	21 (41)	NS
**Mouth**			
**Tongue**			
Fissuring Lambda	6 (5)	1 (2)	≤0.05
**Teeth**			
Normal	59 (48)	32 (64)	≤0.001
Restorations	44 (36)	69 (137)	≤0.001
Missing teeth	17 (14)	35 (70)	≤0.01
**Comparison by Occupation**	**Admin/Sales, % (n)**	**Manufacturing, % (n)**	**P value**
**Mouth**			
**Jaw**			
Jaw symmetry	91 (181)	99 (81)	≤0.05
Jaw -TMJ crepitus with opening	17 (33)	4 (3)	≤0.01
**Hard palate**			
Hard Palate Intact	100 (200)	95 (78)	≤0.01
**Gums**			
Gums - normal	83 (166)	70 (57)	≤0.01
Gum line color abnormal	25 (50)	0 (0)	≤0.001
Gums - gingivitis	5 (9)	20 (16)	≤0.001
Gums - periodontitis	1 (1)	15 (12)	≤0.01
**Tongue**			
Tongue color - red	6 (12)	14 (11)	NS
Tongue shape	51 (102)	90 (73)	≤0.001
Scalloping	20.5 (41)	7.4 (6)	≤0.05
Tongue Size - enlarged	15 (29)	5 (4)	≤0.05

Tongue taste bud distribution and prominence - geographic	3 (5)	9 (7)	≤0.05
**Teeth**			
Normal	47 (93)	23 (19)	≤0.001
Tooth Decay	8 (15)	24 (20)	≤0.001
Teeth-Untreated decay	3 (5)	18 (15)	≤0.001
Teeth painful	1 (2)	12 (10)	≤0.001
Teeth painful - cracked	0 (1)	6 (5)	≤0.01
Restorations - silver/mercury	37 (73)	51 (42)	≤0.05
Missing teeth	21 (42)	52 (42)	≤0.001
Teeth - attrition and abrasions	3 (5)	13 (11)	≤0.001
Missing teeth/restorations	49 (98)	73 (59)	≤0.001

Explanations: Hard palate intact- no cleft palate repair, torus palatinus or other anomalies present.

Supplemental Table S4. Physical Exam finding for Smell, Taste and Sensory Neurological Exam Comparison by Sex, Age, and Occupation

**Smell and Taste**			
**Comparison by Sex**	**Male, % (n)**	**Female, % (n)**	**p value**
**Smell**			
Smell test; odors: chocolate, strawberry, smoke, leather	96 (79)	89 (149)	NS
Abnormal	4 (3)	11 (18)	NS
**Taste**			
Bitter taste - super taster	13 (13)	24 (45)	≤0.01
**Light Touch**			
Lower extremity monofilament, normal	91 (88)	97 (178)	≤0.05
Lower extremity monofilament, left foot diminished	10 (10)	2 (3)	≤0.01
Lower extremity monofilament, right foot diminished	7 (7)	1 (2)	≤0.01
**Comparison by Age**	**Age 18-35, % (n)**	**>35, % (n)**	**p value**
**Taste**			
Bitter strip test, no taste	33 (27)	45 (90)	NS
**Balance**			
Left leg eyes closed no balance	12 (10)	27 (52)	≤0.01
Left leg eyes closed balanced	87 (72)	68 (131)	≤0.001
Right leg eyes closed no balance	11 (9)	24 (46)	≤0.05
Right leg eyes closed balanced	88 (70)	74 (143)	≤0.05
**Vibratory Sense and Monofilament sensation**			
Lower Extremity			
Normal	76 (63)	55 (109)	≤0.01
Left foot diminished vibratory sense	24 (20)	46 (92)	≤0.001
Right foot diminished vibratory sense	24 (20)	46 (91)	≤0.01
Monofilament, diminished	0 (0)	5 (9)	NS
Upper Extremity			
Normal	95 (79)	86 (172)	≤0.05
Left hand diminished vibratory sense	5 (4)	13 (26)	NS
Right hand diminished vibratory sense	5 (4)	13 (27)	≤0.05
**Comparison by Occupation**	**Admin/Sales, % (n)**	**Manufacturing, % (n)**	**p value**
**Taste**			
Taste: No bitter taste	37 (72)	55 (45)	≤0.01
Taste: Some bitter taste	40 (79)	28 (23)	NS
Taste: Super bitter taster	25 (45)	16 (13)	NS

Tasters: Super and some bitter tasters	63 (124)	44 (36)	≤0.01
**Sensory Exam**			
**Vibratory sense**			
Upper Extremity vibratory sense			
Normal	93 (188)	77 (63)	≤0.001
Left hand diminished	5 (11)	23 (19)	≤0.001
Right hand diminished	6 (13)	22 (18)	≤0.001
Lower extremity vibratory sense			
Normal	72 (143)	35 (29)	≤0.05
Left foot diminished	29 (57)	67 (55)	≤0.001
Right foot diminished	28 (56)	67 (55)	≤0.001
**Light Touch**			
Upper extremity			
Monofilament abnormal left hand	0 (0)	4 (3)	≤0.05
Monofilament abnormal right hand	0 (0)	2 (2)	NS
